# LncRNA H19 Suppresses Osteosarcomagenesis by Regulating snoRNAs and DNA Repair Protein Complexes

**DOI:** 10.3389/fgene.2020.611823

**Published:** 2021-01-15

**Authors:** An Xu, Mo-Fan Huang, Dandan Zhu, Julian A. Gingold, Danielle A. Bazer, Betty Chang, Donghui Wang, Chien-Chen Lai, Ihor R. Lemischka, Ruiying Zhao, Dung-Fang Lee

**Affiliations:** ^1^Department of Integrative Biology and Pharmacology, McGovern Medical School, The University of Texas Health Science Center at Houston, Houston, TX, United States; ^2^Department of Obstetrics and Gynecology and Women's Health, Einstein/Montefiore Medical Center, Bronx, NY, United States; ^3^Department of Neurology, Renaissance School of Medicine at Stony Brook University, Stony Brook, NY, United States; ^4^Department of Cell, Developmental and Regenerative Biology, Icahn School of Medicine at Mount Sinai, New York, NY, United States; ^5^The Black Family Stem Cell Institute, Icahn School of Medicine at Mount Sinai, New York, NY, United States; ^6^The Graduate School of Biomedical Sciences, Icahn School of Medicine at Mount Sinai, New York, NY, United States; ^7^Institute of Molecular Biology, National Chung Hsing University, Taichung, Taiwan; ^8^Graduate Institute of Chinese Medical Science, China Medical University, Taichung, Taiwan; ^9^The University of Texas MD Anderson Cancer Center UTHealth Graduate School of Biomedical Sciences, Houston, TX, United States; ^10^Center for Stem Cell and Regenerative Medicine, The Brown Foundation Institute of Molecular Medicine for the Prevention of Human Diseases, The University of Texas Health Science Center at Houston, Houston, TX, United States; ^11^Center for Precision Health, School of Biomedical Informatics and School of Public Health, The University of Texas Health Science Center at Houston, Houston, TX, United States

**Keywords:** osteosarcoma, H19 lncRNA, iPSCs, Li-Fraumeni syndrome, snoRNA, p53

## Abstract

Osteosarcoma is one of the most frequent common primary malignant tumors in childhood and adolescence. Long non-coding RNAs (lncRNAs) have been reported to regulate the initiation and progression of tumors. However, the exact molecular mechanisms involving lncRNA in osteosarcomagenesis remain largely unknown. Li-Fraumeni syndrome (LFS) is a familial cancer syndrome caused by germline p53 mutation. We investigated the tumor suppressor function of lncRNA H19 in LFS-associated osteosarcoma. Analyzing H19-induced transcriptome alterations in LFS induced pluripotent stem cell (iPSC)-derived osteoblasts, we unexpectedly discovered a large group of snoRNAs whose expression was significantly affected by H19. We identified SNORA7A among the H19-suppressed snoRNAs. SNORA7A restoration impairs H19-mediated osteogenesis and tumor suppression, indicating an oncogenic role of SNORA7A. TCGA analysis indicated that SNORA7A expression is associated with activation of oncogenic signaling and poor survival in cancer patients. Using an optimized streptavidin-binding RNA aptamer designed from H19 lncRNA, we revealed that H19-tethered protein complexes include proteins critical for DNA damage response and repair, confirming H19's tumor suppressor role. In summary, our findings demonstrate a critical role of H19-modulated SNORA7A expression in LFS-associated osteosarcomas.

## Introduction

Li-Fraumeni syndrome (LFS) (OMIM #151623) is a rare familial cancer syndrome characterized by early onset of various tumors, soft-tissue sarcomas, osteosarcomas, breast cancers, brain tumors, adrenocortical carcinomas, and leukemia (Zhou et al., 2017). Germline mutations in the p53 tumor suppressor gene are responsible for LFS. Patient-derived iPSCs have been used to model various diseases (Lee et al., 2009b; Carvajal-Vergara et al., 2010; Itzhaki et al., 2011; Yagi et al., 2011; Mulero-Navarro et al., 2015; Gingold et al., 2016; Liu et al., 2018; Zhu et al., 2018). The development of various refined differentiation protocols utilizing induced pluripotent stem cells (iPSCs) has enabled the production of large quantities of differentiated cells from individual patients. In our previous studies, using a LFS patient-derived iPSC model, we demonstrated the tumor suppressor role of lncRNA H19 and the oncogenic role of SFRP2 during the formation of osteosarcoma in LFS patients (Lee et al., 2015; Lin et al., 2017b; Kim et al., 2018; Zhou et al., 2018). Although H19-mediated tumor suppression has been demonstrated in LFS-associated osteosarcomagenesis, the underlying mechanisms of its tumor suppressor activity remain unclear.

Long non-coding RNAs (lncRNAs) represent a class of nonprotein-coding RNAs longer than 200 nucleotides (Sanchez Calle et al., 2018). Despite not being translated into proteins, lncRNAs are important regulators of diverse biological processes and pathologies. LncRNAs are proposed to function via multiple mechanisms, including co-transcriptional regulation, bridging proteins and chromatin, offering cytoplasmic scaffolding, pairing with other RNAs, and serving as molecular decoys (Ulitsky and Bartel, 2013).

One of the most studied lncRNAs is the imprinted lncRNA H19, located on human chromosome 11 and expressed exclusively from the maternal allele (Gabory et al., 2009). Numerous functional studies have assessed the role of H19 in the pathogenesis of human cancers and yielded conflicting results. On one hand, H19 is a precursor of miR-675 (Keniry et al., 2012) as well as a “molecular sponge” for soaking up microRNAs let-7 (Kallen et al., 2013; Li et al., 2020) and miR-138 (Liang et al., 2015), supporting a role in promoting cancer cell proliferation and migration. On the other hand, *in vivo* mouse (Yoshimizu et al., 2008) and LFS iPSC-derived osteoblast (Lee et al., 2015) studies demonstrated that H19 displays a tumor-suppressive effect. Evidence of numerous human tumors displaying either overexpression or lack of expression of H19 (Hao et al., 1993; Lustig-Yariv et al., 1997; Yoshimizu et al., 2008; Lee et al., 2015; Li et al., 2020) suggests the possibility of context-dependent oncogenic and tumor-suppresive roles.

Small nucleolar RNAs (snoRNAs) are small non-coding RNAs 60–300 nucleotides in length, primarily located in the nucleolus. The main functions of snoRNAs are to assist with post-transcriptional modification and maturation of ribosomal RNAs (rRNAs), small nuclear RNA (snRNAs), and other cellular RNAs. SnoRNAs can interact with RNA binding proteins to form small nuclear ribonucleoproteins. SnoRNAs are divided into two classes according to their catalytic activity: C/D box snoRNAs, catalyzing 2-O-ribose methylation; and H/ACA box snoRNAs, catalyzing pseudouridylation (Bachellerie et al., 2002). Emerging evidence indicates that snoRNAs are widely involved in various cancer-related signaling pathways. For example, SNORA42 is overexpressed in non-small cell lung cancer (NSCLC) and plays an oncogenic role through suppressing p53 function and/or expression (Mei et al., 2012). SNORD76 leads to the activation of the WNT/β-Catenin pathway to promote hepatocellular carcinoma (HCC) tumorigenicity (Wu et al., 2018).

The DNA damage/repair response plays a key role in maintaining genome integrity and stability, and its dysfunction girds the development and progression of various cancer types. Recently, studies revealed that lncRNA also regulates DNA damage response and DNA repair through the ATM/ATR (Wan et al., 2013), p53 regulatory network (Hu et al., 2018), and DNA double-strand break repair pathway (Gazy et al., 2015). However, it remains unclear whether H19 is actively involved in DNA damage/repair response.

Our current study reveals that H19 functions as a tumor suppressor through negatively regulating the expression of oncogenic SNORA7A in the osteoblast context. Furthermore, by exploring its interactions with multiple essential DNA damage/repair response factors, we demonstrate how H19 executes its tumor-suppressive role.

## Materials and Methods

### Cell Cultures

U2OS cells were maintained in DMEM (Invitrogen, USA) supplemented with 10% FBS and 1% penicillin/streptomycin. iPSCs were maintained on Matrigel (Corning, USA)-coated plates in StemMACS™ iPS-Brew XF medium (MiltenyiBiotec, USA). All cells were cultured in a humidified incubator at 37°C and 5% CO2 and were tested to exclude mycoplasma contamination.

### Differentiation of iPSCs to Mesenchymal Stem Cells (MSCs) and Then to Osteoblasts

*In vitro* differentiation of LFS iPSCs to MSCs was performed by a PDGF-AB-based method described previously (Lian et al., 2007; Zhou et al., 2018). Appropriately differentiated CD105+/CD166+/CD24- MSCs were plated in a 6-well plate at a density of 2 × 10^4^ cells per well and cultured in an osteogenic differentiation medium (α-MEM supplemented with 10% FBS, 10 mM β-glycerol phosphate, 200 μM ascorbic acid, and 0.1 μM dexamethasone) to induce osteogenic differentiation as previously described (Lee et al., 2015).

### RNA Isolation and RNA-Sequencing

LFS osteoblasts with ectopic H19 expression and vector control osteoblasts were collected and compared for H19-mediated tumor suppressor effects. Total mRNA was isolated using TRIzol reagent (Thermo Fisher Scientific, USA) according to the manufacturer's instructions. All RNA sample preparation and RNA-sequencing (RNA-seq) data analyses were performed as described previously (Lee et al., 2015). RNA was aggregated from biological triplicate experiments prior to running as a single sample for RNA-seq. The FPKM (fragments per kilobase of exon model per million reads mapped) of genes, lncRNAs, snoRNAs, and miRNAs were calculated and summarized in Supplementary Table 1.

### Enrichr Analysis

GO Biological Process (GO_BP) and Wikipathway analyses were performed using Enrichr (https://amp.pharm.mssm.edu/Enrichr/) (Chen et al., 2013; Kuleshov et al., 2016) to identify enriched biological processes and pathways in H19-expressing LFS osteoblasts. Processes and pathways enriched with a *p* < 0.05 were considered significant. These genes were analyzed in Enrichr using Wikipathway and GO_BP pathway datasets to identify enriched GO terms, pathways, and functions. The TRANSFAC and JASPAR PWMs databases of transcription factors were used in Enrichr to identify transcription factors positively and negatively correlated with SNORA7A expression.

### snoRNA Analysis

To determine pathological functions in osteosarcomagenesis significantly associated with SNORA7A, we identified genes positively and negatively correlated with SNORA7A expression in the TCGA-SARC dataset from SNORic (snoRNA in cancers; http://bioinfo.life.hust.edu.cn/SNORic/basic/) (Gong et al., 2017). Correlation of SNORA7A and mRNA expression of significant genes was run at default settings. Clinical analysis of 5-year survival in different cancers was analyzed from TCGA.

### Plasmid Construction

A modified 1x streptavidin-binding RNA aptamer (S1m) (Leppek and Stoecklin, 2014) was synthesized (Integrated DNA Technologies, USA) from DNA oligo pairs (S1m_Forward: AATTGgtagaaaATGCGGCCGCCGACCAGAATCATGCAAGTGCGTAAGATAGTCGCGGGTCGGCGGCCGCATctgctgggG; S1m_Reverse: AATTCcccagcagATGCGGCCGCCGACCCGCGACTATCTTACGCACTTGCATGATTCTGGTCGGCGGCCGCATtttctacC), annealed, and ligated into the multiple cloning sites of the tetracycline-inducible TetO-FUW vector (Lee et al., 2015) 4 times sequentially. The TetO-FUW vector containing 4×streptavidin-binding RNA aptamer was named TetO-FUW-4S1m. H19 was then cloned into the N terminal side of 4S1m to form TetO-FUW-4S1m-H19. For the SNORA7A expression construct, the synthesized full-length SNORA7A (Ensembl Transcript ID: ENST00000384765.1) was cloned into the pLKO.pig plasmid (Lee et al., 2012a,b) within EcoRI-AgeI cloning sites and confirmed by Sanger sequencing.

### qRT-PCR

Total RNA was isolated using TRIzol reagent (Thermo Fisher Scientific, USA) and snoRNA was isolated using mirVana miRNA isolation kit (Thermo Fisher Scientific, USA) according to the manufacturer's instructions. The qRT-PCR primers for GAPDH (internal control), BGLAP, MEPE, FGF23, H19, and SNORA7A were described previously (Lee et al., 2015; Zhang et al., 2017).

### Virus Packaging and Infection

TetO-FUW-4S1m or TetO-FUW-4S1m-H19 plasmids were co-transfected with packaging plasmids psPAX2 (Addgene, plasmid # 12260) and pMD2.G (Addgene, plasmid # 12259) into HEK-293T cells, and the virus collected from the cell culture medium 48 hours later. Osteosarcoma cell line U2OS was infected with viral particles together with the M2rtTA virus (Addgene, plasmid # 20342), which was similarly produced in HEK-293T cells, in the presence of 8ug/ml polybrene (Sigma-Aldrich, USA). Thirty-six hours post-infection, U2OS cells were treated with 1μg/ml doxycycline (Sigma-Aldrich, USA) for 24 hours to induce the expression of 4S1m or 4S1m-H9.

### *In vitro* Anchorage-Independent Growth (AIG) Assay

AIG assay was performed as described previously (Lee et al., 2015). Briefly, LFS MSCs were transduced with H19 and/or SNORA7A. 10,000 LFS MSCs were mixed with 0.5% UltraPure low-melting-point agarose (Thermo Fisher Scientific, USA) and cultured in an osteogenic differentiation medium for 1 month. Colonies (≥50 μm) were counted under a Leica microscope DMi8.

### H19-Interacting Protein Complex Purification and Mass Spectrometry Analysis

H19-interacting proteins were purified via the streptavidin-binding aptamers S1m as described previously (Leppek and Stoecklin, 2014). Briefly, S1m-H19 expressing U2OS cells were resuspended in 500 μl ice-cold lysis buffer (20 mM Tris-HCl (pH 7.5), 150 mM NaCl, 1.5 mM MgCl_2_, 2 mM DTT, 2 mM RNase inhibitor, and complete protease inhibitors cocktail). Lysates were subjected to centrifugation for 5 min at 12,000 rpm at 4°C. The supernatants were incubated with streptavidin agarose beads (Thermo Fisher Scientific, USA) overnight at 4°C under rotation. The streptavidin agarose beads were then washed five times for 5 min at 4°C with wash buffer [20 mM Tris-HCl (pH 7.5), 300 mM NaCl, 5 mMMgCl_2_, and 2 mM DTT]. The 4S1m-H19 associated protein complex samples were subjected to SDS-PAGE electrophoresis and micro-liquid chromatography/tandem mass spectrometry as described previously (Lee et al., 2007, 2009a, 2012a). Mass spectrometry analysis results are summarized in Supplementary Table 2.

### Statistical Analyses

Results were presented as mean ± standard error of the mean (SEM). Error bars in figures represent SEM. Differences between two groups were examined by the two-tailed unpaired Student *t*-test. *p < 0.05; **p < 0.01; and ***p < 0.001.

### Data Availability

The data supporting the findings are available within the manuscript text, figures, and Supplementary Tables 1, 2. The RNA-seq data are available at the sequencing read archive (SRA) under accession number PRJNA673185.

## Results

### H19 Modulates snoRNA Expression in LFS Osteoblasts

**Figure 1:**
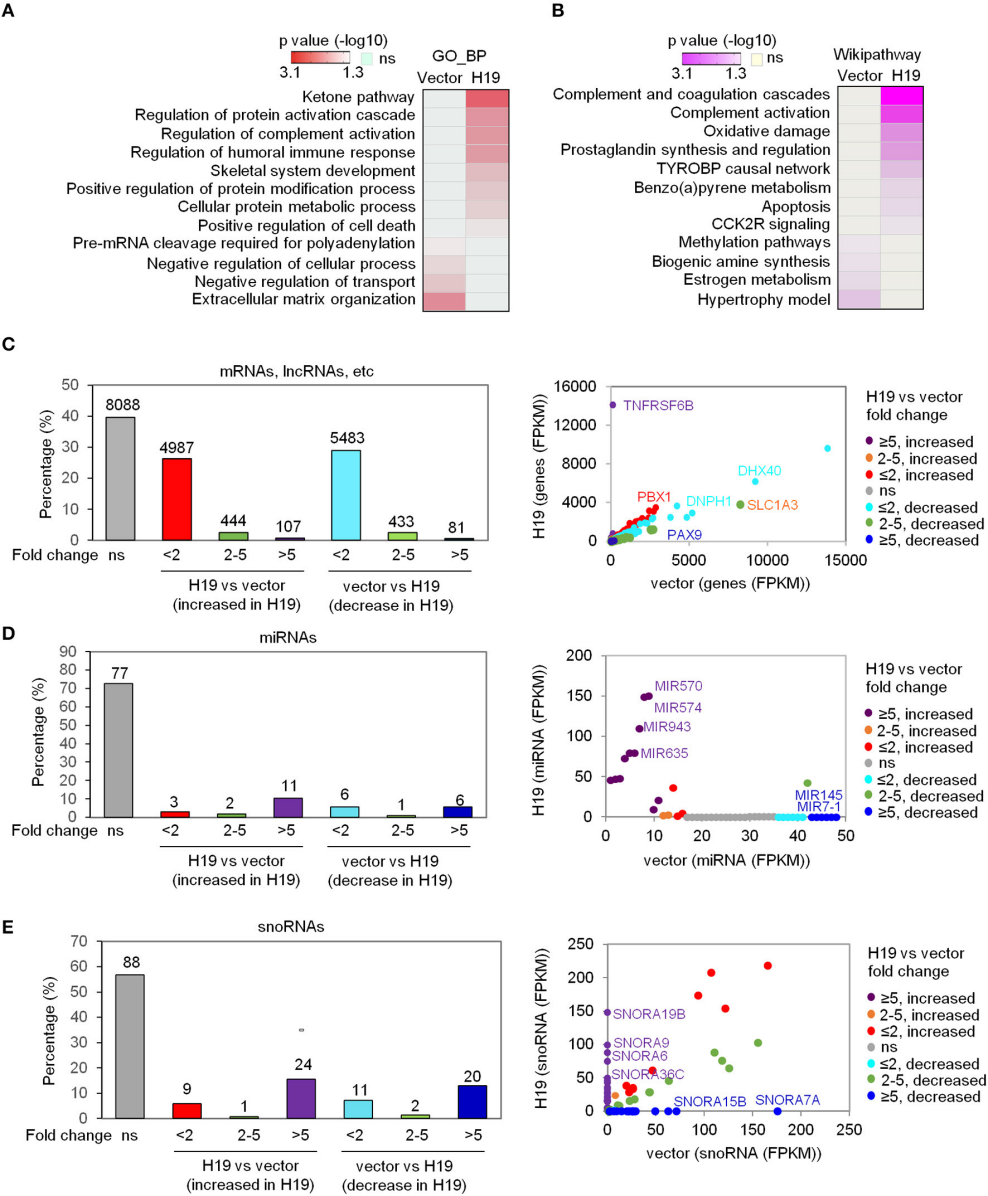
H19 significantly affects miRNA and snoRNA expression. **(A,B)** Heatmaps show the GO_BP **(A)** and Wikipathway **(B)** categories enriched in either H19-expressing LFS osteoblasts (right column) or vector control LFS osteoblasts (left column) compared with each other based on mRNA expression of respective samples. Colors represent *p* values of enrichment. **(C-E)** H19 overexpression induces small changes in overall mRNA expression but large changes in miRNA and snoRNA expression in LFS osteoblasts. The percentage of mRNAs, lncRNAs, etc. **(C)**, miRNAs **(D)**, and snoRNAs **(E)** with different fold-changes (<2, 2-5, and >5 increase or decrease) are shown in bar plots. RNA expression between H19-overexpressing and vector control cells is presented in a scatter plot (right). Dot colors are used to represent the fold change of RNA expression. ns (not significant) indicates mRNAs, lncRNAs, miRNAs, snoRNAs, etc. with fold-change <2 and/or FPKM<1. The RNA-seq experiment was done with one sample pooled from 3 biological replicates. miRNAs and snoRNAs are defined based on the miRbase and snoDB database, respectively. “mRNA, lncRNA, etc.” indicates all transcripts except miRNAs and snoRNAs.

To explore the suppressive effects of H19 on osteosarcomagenesis, we analyzed the genome-wide transcriptomes of LFS iPSC-derived osteoblasts by RNA-seq following ectopic overexpression of H19. The RNA-seq experiment was performed with either vector- or H19-expressing LFS osteoblasts (*n* = 1) after pooling RNA from biological triplicate experiments. Transcriptome analysis confirmed ectopic H19 overexpression and identified a set of significantly altered genes [107 upregulated genes (FPKM ≥1 and fold change ≥5) and 81 downregulated genes (FPKM ≥1 and fold change ≥5)]. These H19-regulated genes were analyzed for enriched Gene Ontology biological processes (GO_BP) and Wikipathways using the comprehensive gene set enrichment analysis web tool Enrichr. GO_BP analyses showed that the GO_BPs enriched in H19-expressing LFS osteoblasts compared with vector-expressing (H19-depleted) LFS osteoblasts included ketone pathway, regulation of complement activation, skeletal system development, and positive regulation of cell death. In contrast, H19-depleted LFS osteoblasts demonstrated enrichment of GO_BPs including pre-mRNA cleavage required for polyadenylation, negative regulation of the cellular process, negative regulation of transport, and extracellular matrix organization (Figure 1A). Wikipathway analyses indicated that H19-overexpressing LFS osteoblasts were enriched for genes in the complement and coagulation cascade, complement activation, oxidative damage, prostaglandin synthesis, and regulation, TYROBP causal network, benzopyrene metabolism, apoptosis, and CCK2R signaling. In contrast, genes enriched in H19-depleted LFS osteoblasts were involving in methylation pathways, biogenic amine synthesis, estrogen metabolism, and hypertrophy model (Figure 1B).

In analyzing the transcriptome data, we classified RNA alterations into three groups by their fold-change: less than 2, between 2 and 5, and higher than 5. We unexpectedly found that a much larger share of miRNAs and snoRNAs increased in expression following H19 expression in LFS osteoblasts compared with protein-coding mRNAs, lncRNAs, etc. (i.e all transcripts except miRNAs and snoRNAs). H19 restoration in LFS osteoblasts led to a more than 5-fold change (increased or decreased) in expression in 0.96% of protein-coding mRNAs, lncRNAs, etc. with the majority of mRNAs and lncRNAs changing <2-fold (FPKM <1) (Figure 1C). In contrast, 16% of miRNAs and 28.4% of snoRNAs changed greater than 5-fold in expression (Figures 1D,E). Scatter plots comparing miRNA and snoRNA expression between H19-expressing and vector control LFS osteoblasts showed that miRNAs (e.g., MIR570, MIR574, MIR943, MIR635, MIR7-1, and MIR145) and snoRNAs (e.g., SNORA6, SNORA7A, SNORA9, SNORA15B, SNORA19B, and SNORA36C) were significantly altered in H19-expressing LFS osteoblasts (Figures 1D,E). These findings suggest that H19 suppresses osteosarcomagenesis in LFS patients by primarily altering the expression of miRNAs and snoRNAs rather than mRNAs.

### SnoRA7A Is an onco-snoRNA Involved in Tumor Progression and Associated With Poor Prognosis

**Figure 2:**
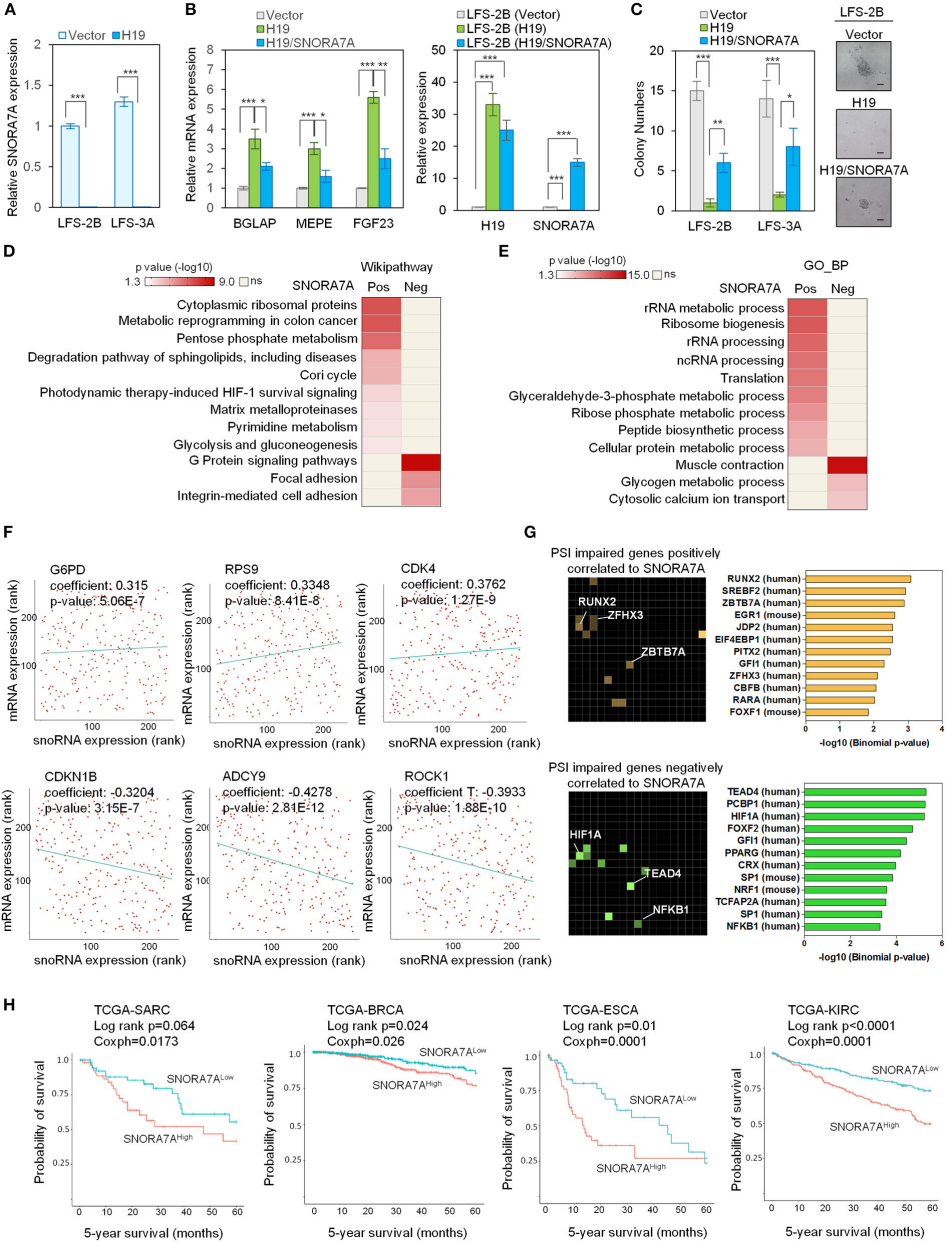
Systems analyses of the oncogenic role of SNORA7A from TCGA datasets. **(A)** Ectopic expression of H19 downregulates SNORA7A expression in LFS osteoblasts. qRT-PCR data are represented as mean ±SEM; *n* = 3 biological replicates; statistical significance is determined using two-tailed Student's *t*-test; ***p < 0.001. **(B)** Left panel, ectopic expression of H19 increases osteogenic gene expression in LFS osteoblasts; in contrast, restoration of SNORA7A expression impairs H19-upregulated osteogenic gene expression in H19-transduced LFS osteoblasts. Right panel, qRT-PCR results demonstrate the expression of H19 and SNORA7A upon their ectopic expression for assays in the left panel. qRT-PCR data are represented as mean ± SEM; *n* = 3 biological replicates; statistical significance is determined using two-tailed Student's *t*-test; *p < 0.05; **p < 0.01; ***p < 0.001. **(C)** AIG assay for *in vitro* tumorigenicity demonstrates that H19 impairs the colony numbers of LFS osteoblasts and SNORA7A expression rescues the H19-suppressed tumorigenicity of LFS osteoblasts. H19 or H19/SNORA7A-transduced LFS osteoblasts were grown for 1 month and then assayed. Positive colonies are considered those larger than 50μm diameter. Data are represented as mean ± SEM; *n* = 6 biological replicates; statistical significance is determined using two-tailed Student's *t*-test; *p < 0.05; **p < 0.01; ***p < 0.001. **(D,E)** Genes positively (*n* = 780) and negatively (*n* = 945) correlated with SNORA7A expression in TCGA-SARC are identified. Pathway analysis by Wikipathway **(A)** and GO_BP **(B)** is performed on these gene sets using Enrichr to identify pathways significantly enriched or depleted (*p* ≤ 0.05) in association with SNORA7A expression. **(F)** Scatterplots of G6PD, RPS9, CDK4, CDKN1B, ADCY9, and ROCK1 mRNA expression correlation with SNORA7A expression. **(G)** Genes whose abnormal splicing (including exon skip, mutually exclusive splicing, or intron retention) by PSI was positively (*n* = 91, upper panel) or negatively (*n* = 182, lower panel) correlated with SNORA7A expression in sarcomas are identified by TRANSFAC and JASPAR PWWMs in Enrichr. PSI: percent spliced in index. **(H)** High SNORA7A expression is associated with poor cancer survival. Five-year overall survival is analyzed according to SNORA7A expression from TCGA in various cancers. SARC: sarcoma. BRCA: breast invasive carcinoma. ESCA: esophageal carcinoma. KIRC: kidney renal clear cell carcinoma.

Among the identified H19-regulated miRNAs and snoRNAs, SNORA7A was chosen for further study in light of its previously recognized role in controlling the self-renewal of MSCs (Zhang et al., 2017). To validate our transcriptome results, we ectopically expressed H19 in LFS iPSC-derived osteoblasts and found that H19 significantly inhibits SNORA7A expression (Figure 2A). H19-induced osteogenic gene expression (e.g., BGLAP, MEPE, and FGF23) was inhibited by SNORA7A (Figure 2B), indicating that SNORA7A inhibits osteogenesis. To investigate whether the inhibition of SNORA7A plays a role in H19-mediated tumor suppression, we performed an *in vitro* AIG assay and found retarded clonal growth of H19-transduced LFS osteoblasts upon SNORA7A ectopic expression (Figure 2C). These results suggest that SNORA7A is negatively regulated by H19, and that SNORA7A functions as an onco-snoRNA by antagonizing H19 tumor suppressor function.

To further explore the role of SNORA7A in tumorigenesis, we analyzed the TCGA-SARC dataset and identified 780 and 945 protein-coding genes whose mRNA levels were positively and negatively correlated with SNORA7A expression, respectively. Wikipathway analysis indicated that genes positively correlated with SNORA7A expression are mainly involved in cytoplasmic ribosomal protein function and cell metabolism (e.g., pentose phosphate metabolism, Cori cycle, pyrimidine metabolism, and glycolysis and gluconeogenesis), while genes negatively correlated with SNORA7A expression are associated with focal adhesion, integrin-mediated cell adhesion, and G protein signaling pathways, the latter of which is involved in osteoblast differentiation (Wu et al., 2010) (Figure 2D).

Similarly, GO_BP analysis demonstrated that genes positively correlated with SNORA7A expression are involved in rRNA metabolic process and processing, ribosome biogenesis, translation, glyceraldehyde-3-phosphate metabolic process, and ribose phosphate metabolic process, while genes negatively correlated with SNORA7A expression are associated with glycogen metabolic process and cytosolic calcium ion transport (Figure 2E). The molecules controlling pentose phosphate metabolism (G6PD), protein translation (RPS9), and cell cycle progression (CDK4) were positively correlated with SNORA7A expression. In contrast, cyclin-dependent kinase inhibitor (CDKN1B), adenylyl cyclase (ADCY9), and downstream effector of Rho (ROCK1) were negatively correlated with SNORA7A expression (Figure 2F). These systems analyses suggest that SNORA7A may transcriptionally and/or post-transcriptionally regulate multiple oncogenic features, including cellular metabolism, ribosome biogenesis, cell cycle, etc., culminating in osteosarcoma development in LFS patients.

The regulation of RNA splicing is increasingly recognized to be an essential mechanism underlying cancer development, and dysregulation of RNA splicing machinery has been found to contribute to tumorigenesis in various human cancers, including glioma, lymphoma, and breast cancer (David et al., 2010; Hsu et al., 2015; Koh et al., 2015). Given the well-recognized potential for snoRNAs to regulate pre-mRNA splicing (Falaleeva et al., 2016; Liang et al., 2019), we investigated the effects of SNORA7A levels toward potentially pathological mRNA splicing events including skipped exons, mutually exclusive alternative splicing, and intron retention. The percent spliced in index (PSI) was calculated for all identified genes in the TCGA-SARC dataset to identify genes whose alternative splicing was correlated with SNORA7A expression. These genes were then mapped to transcription factors using TRANSFAC and JASPAR analysis. Genes whose abnormal splicing was positively correlated to SNORA7A expression were mainly downstream targets of transcription factors such as the osteoblastic lineage regulator RUNX2 (Komori, 2019) and the tumor suppressors ZBTB7A (Liu et al., 2014) and ZFHX3 (Hu et al., 2019). Moreover, genes whose abnormal splicing was negatively correlated to SNORA7A expression were downstream targets of transcription factors such as the tumor angiogenesis regulator HIF1A (Koukourakis et al., 2002), the Hippo pathway transcription factor TEAD4 (Lin et al., 2017a; Shi et al., 2017) and the tumorigenic transcription factor NF-KB1 (Concetti and Wilson, 2018) (Figure 2G). These results imply that oncogenic effects of SNORA7A occur through regulation of RNA splicing of tumor suppressor genes and oncogenes.

We next analyzed the expression of SNORA7A in multiple cancers using TCGA datasets and correlated its expression with patient survival data. The five-year overall survival analyses showed that high SNORA7A levels were significantly associated with poorer survival in multiple cancer types including sarcoma (SARC, Log-rank *p* = 0.064, coxph = 0.017), breast cancer (BRCA, Log-rank *p* = 0.024, coxph = 0.026), esophageal carcinoma (ESCA, Log-rank *p* = 0.01, coxph = 0.0001), and kidney carcinoma (KIRC, Log-rank *p* ≤ 0.0001, coxph = 0.0001) (Figure 2H). Taken together, these results suggest a potential role of SNORA7A in promoting cancer progression and emphasize that H19 tumor suppressor functions may be mediated through the repression of SNORA7A expression.

### H19 Potentially Interacts With DNA Damage/Repair Response Protein Complexes

**Figure 3:**
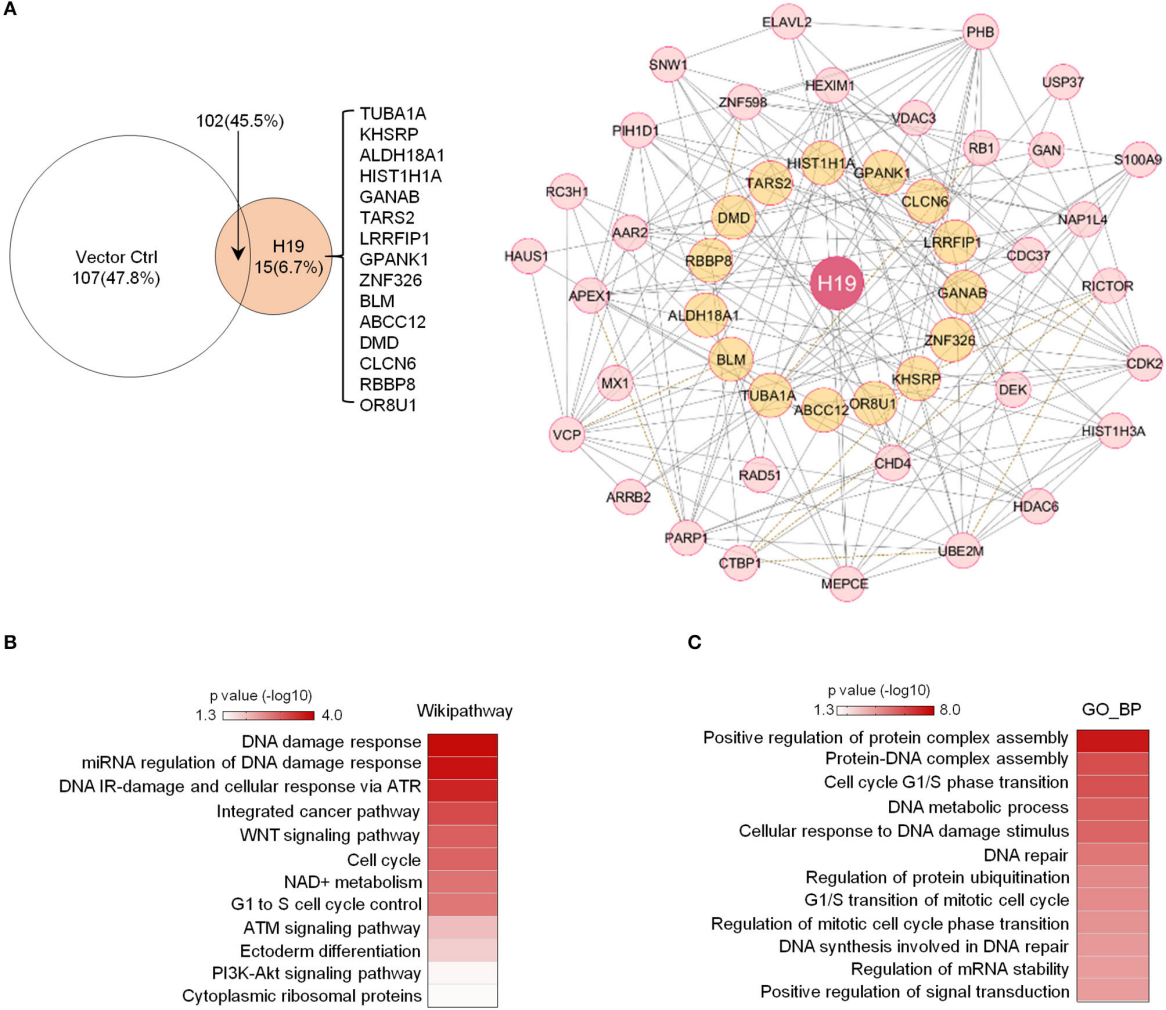
Potential H19 interacting proteins are primarily involved in DNA damage/repair response. **(A)** Left panel: Mass spectrometry analysis of TetO-FUW-4S1m-H19 streptavidin pull-down proteins identifies 15 unique H19-interacting proteins. TetO-FUW-4S1m vector is used as an S1m pull-down negative control (left). Right panel: The biological protein-protein interaction network based on H19-interacting proteins (orange, *n* = 15) and expanded to include genes (pink, *n* = 31) connected to at least two H19-interacting proteins is constructed in Cytoscape using the Biogrid human database. A solid line indicates physical interactions. A dashed line indicates genetic interactions. **(B,C)** Heatmaps representing p-values of enriched genesets (*p* ≤ 0.05) among the H19-interacting protein complex genes (*n* = 47) by Wikipathway **(B)** and GO_BP **(C)** analyses.

Previous studies revealed that H19 is capable of interacting with EZH2 (Luo et al., 2013) to promote WNT/β-Catenin activation and subsequent downregulation of E-cadherin and that H19 also associates with KSRP (Giovarelli et al., 2014) to control mRNA decay. However, the protein complexes tethered by H19 in osteosarcoma remain largely unknown. To elucidate H19-associated protein complexes and gain insights into the potential biological functions controlled by the H19 interactome, we ligated H19 with an optimized 4 × streptavidin-binding RNA aptamer (S1m) and expressed H19-S1m in U2OS cells. H19-bound protein complexes could then be pulled down with streptavidin beads and identified by mass spectrometry (Supplementary Table 2). Compared with the S1m vector control, S1m-H19 pull-down identified 15 putatively interacting cellular proteins: TUBA1A, KHSRP, ALDH18A1, HIST1H1A, GANAB, TARS2, LRRFIP1, GPANK1, ZNF326, BLM, ABCC12, DMD, CLCN5, RBBP8, and OR8U1 (Figure 3A, left panel).

We applied the Biogrid human database to identify proteins previously demonstrated to bind to at least two of these 15 potential H19-binding proteins. This bioinformatics approach yielded 47 proteins strongly suspected to be connected to the 15 H19-associated proteins (Figure 3A, right panel). Wikipathway and GO_BP analyses revealed that these putative H19-associated protein complexes mainly function in DNA damage response, DNA repair, the ATM/ATR signaling pathway, the WNT signaling pathway, and the cell cycle (Figures 3B,C), emphasizing the potential role of H19 in regulating DNA damage response and repair. Particularly, BLM, an ATP-dependent DNA helicase, participates in DNA replication and DNA repair by modulating DNA double-strand break resection (Gravel et al., 2008). Mutations in the BLM gene are associated with Bloom syndrome, which carries a greatly increased risk of cancers including squamous cell carcinoma, leukemia, lymphoma, and gastrointestinal cancer (Lin et al., 2017b). Another H19-interacting protein is RBBP8 (also known as CtIP), an endonuclease that cooperates with the MRE11-RAD50-NBN (MRN) complex in DNA-end resection, the first step of homologous recombination (HR)-mediated double-strand break repair. RBBP8 mutations are commonly detected in various human cancer cell lines (You and Bailis, 2010). Inactivation of one *RBBP8* allele predisposed mice to multiple types of cancers (e.g., lymphoma) suggesting that RBBP8 functions as a tumor suppressor (Chen et al., 2005). Based on these findings, we speculate that H19 tethers DNA repair proteins to other functional factors and helps maintain genomic integrity by suppressing oncogenic events such as DNA double-stranded breaks.

## Discussion

The essential biological functions of non-coding RNAs (e.g., miRNAs, snoRNAs, and lncRNAs) and their pathologic roles in tumorigenesis are becoming increasingly appreciated. In our previous work, we reported that H19, a lncRNA, is downregulated by mutant p53s (mutp53s) in LFS iPSC-derived osteoblasts and modulates osteoblastic differentiation and oncogenic repression through the imprinted gene network (IGN) (Gabory et al., 2009; Lee et al., 2015). While H19 has been demonstrated in various contexts to act as either a tumor suppressor or an oncogene, our current work further demonstrates the important regulatory role of H19 in osteosarcoma initiation and progression.

We discovered considerable H19-mediated regulation of snoRNAs. We confirmed that SNORA7A, a snoRNA known to promote MSC proliferation and self-renewal (Zhang et al., 2017), is suppressed by H19 and that restoration of SNORA7A expression impaired H19-mediated osteogenesis and tumor suppression. These findings suggest that SNORA7A functions as an onco-snoRNA in LFS-associated osteosarcoma. SNORNA7 expression in TCGA datasets is associated with numerous oncogenic pathways (ribosome biogenesis, pentose phosphate pathway, glycolysis, and HIF signaling) and RNA splicing events, suggesting a link between snoRNA expression and oncogenic events in clinical tumors.

p53 mutations have been widely linked to increased tumor oncogenesis through both gain-of-function interactions, for example by promoting HIF1A-regulated angiogenic genes (Amelio et al., 2018), and loss-of-function mutations, for example by abrogating wild-type p53 repression of FBL expression, culminating in increased ribosome biogenesis (Marcel et al., 2013). Upregulation of SNORA7A by mutp53-mediated H19 suppression provides yet another mechanism for mutp53 downstream pathways to cooperate in promoting bone malignancies.

In addition, mass spectrometry analysis of potential H19-interacting proteins revealed multiple DNA damage response and repair molecules directly associated with H19. H19 was previously proposed to regulate DNA damage response (Zheng et al., 2016; Zhu et al., 2017; Ma et al., 2018; Cheng et al., 2019), but the underlying mechanisms have yet to be defined. Our identification of potential interactions between H19 and BLM as well as RBBP8, both of which are recognized to regulate DNA damage response and repair, may provide another angle to elucidate these mechanisms. Importantly, gene mutations in both BLM and RBBP8 are associated with increased risks of tumor formation, including osteosarcoma. The evidence of physical interactions between H19 and DNA damage response genes leads us to speculate that H19 indirectly maintains genomic integrity, explaining its tumor-suppressor activity.

Finally, H19 expression was significantly increased in cancer cells treated with chemotherapeutic drugs (e.g., doxorubicin and cisplatin) (Zheng et al., 2016; Zhu et al., 2017). We speculate that upregulation of H19 following DNA damage events occurs in order to accelerate the repair process and prevent oncogenic effects of DNA damage.

In conclusion, our study indicated that lncRNA H19 plays a vital regulatory role in inhibiting osteosarcomagenesis and provides mechanistic insights for improving our understanding of H19-mediated tumor suppression in LFS patient-associated osteosarcomas.

## Data Availability Statement

The transcriptome data has been deposited into the sequencing read archive (SRA; PRJNA673185).

## Author Contributions

AX, M-FH, DZ, BC, DW, C-CL, and RZ conducted all experiments. AX, M-FH, DZ, DW, C-CL, IL, RZ, and D-FL conceived and designed the study and interpreted results. AX, M-FH, DZ, JG, DB, RZ, and D-FL wrote the manuscript. All authors contributed to the article and approved the submitted version.

## Conflict of Interest

The authors declare that the research was conducted in the absence of any commercial or financial relationships that could be construed as a potential conflict of interest.
